# Numerical analysis of MHD axisymmetric rotating Bodewadt rheology under viscous dissipation and ohmic heating effects

**DOI:** 10.1038/s41598-022-13676-2

**Published:** 2022-06-16

**Authors:** M. Awais, Marium Bibi, Aamir Ali, M. Y. Malik, Kottakkaran Sooppy Nisar, W. Jamshed

**Affiliations:** 1 Department of Mathematics, COMSATS University Islamabad, Attock Campus, Kamra Road, Attock, 43600 Pakistan; 2 Department of Mathematics, College of Sciences, King Khalid University, Abha, 61413 Saudi Arabia; 3 Department of Mathematics, College of Arts and Sciences, Prince Sattam Bin Abdulaziz University, Wadi Al Dawaser, 11991 Saudi Arabia; 4 Department of Mathematics, Capital University of Science and Technology (CUST), Islamabad, 44000 Pakistan

**Keywords:** Engineering, Nanoscience and technology

## Abstract

In present research manuscript, analysis is presented for the influences of heat transition in a bodewadt flow over a penetrable disk numerically. Estimation parameters in current mathematical flow model include magnetic field parameter $(0.1 \le M \le 1.2),$ wall suction $(1.7 \le A \le 6.7),$ prandtl number $(0.2 \le \Pr \le 5.0),$ heat generation/absorption ${( - }0.9 \le Q \le 3.6),$ eckert number $(0 \le Ec \le 1.2),$ variable viscosity ${( - 100} \le \theta_{e} \le 100)$ and thermal conductivity ${(0} \le \varepsilon \le 4.1).$ The repercussions of joule heating, wall suction, heat generation & absorption, magnetic field, viscous dissipation accompanying with variable characteristics of the fluid are also examined as well. Kinetics of viscous fluid with variable characteristics of fluid having solid body rotation over a permeable disk (having cylindrical geometry) are analyzed. We transformed the governing equations of heat transfer (accompanied by variable properties) and fluid motion in to self-similar non-dimensional differential equations by using the Von-Karman variables which are then further analyzed numerically by utilizing Adams Bashforth method. For a physical insight, results are manifested to scrutinize the behavior of velocity and temperature profiles for different emerging parameters graphically. Moreover, the values of nusselt number & skin friction co-efficient are also computed and physically explicated for the assorted parameters. Outcomes of current investigations are compared with prior work, to ensure the authenticity of the numerical method, and strong agreement is noted.

## Introduction

Three-dimensional flow problems produced by virtue of a rotating viscous fluid are experienced in a number of engineering-relevant, product-designing, industrial and scientific products and procedures for instance centrifuges, viscometers, jet engines, turbines, mixing in chemical chambers, vacuum cleaners, disk drives of computers and turbo-machinery etc. A classicistic problem in the field of fluid mechanics is the problem of fluid flow that is generated by a disk which possesses large diameter and is revolving about it’s origin under the influences of constant angular velocity. Many years ago, Von-Karman^1^ composed and resolved this problem. Anyone doesn’t visualized an accurate circular flow close to the disk in this fluid problem since there is not applied pressure gradient that supplied the radial component of acceleration inwards and, as a consequence, fluid scrolled outside. Von-Karman^1^ who was capable to transform the system of Navier-Stoke’s equation into a set of ordinary differential equations with the assistance of appropriate similarity transformations. Cochran^2^ noted some imprecisions in the solution of Von-Karman’s problem and then developed an asymptotic solution of that problem. Ackroyd^3^ elongated the problem of Von-Karman by accounting the impacts of injection/suction and illustrated series solution that comprises of the exponentially decomposing expressions. A similar problem is originated when the fluid revolves under the influences of constant angular velocity over a motionless plane is second fascinating problem. On the contrary, the fluid particles close to the disk moves in the direction of axis of rotation since it’s circumferential constituent of velocity is decelerated under the similar pressure gradient. Tabassum and Mustafa^4^ modeled numerically the heat transfer and partial slip flow of reiner-rivlin non-newtonian fluid generated by a revolving disk. A distinctive illustration made by researchers in this regard is given in references^5–10^. Contrary to Von-Karman^1^ problems, Bodewadt^11^ demonstrated that problem in 1940 by constructing boundary layer conditions. In Bodewadt’s flow, fluid particles around the disk under the impact of radial pressure gradient directed to proceed inside towards the rotational axis where fluid is in the condition of stiff-body rotation. Bodewadt flow is monitored in a tea cup when rotation in the fluid is urged by continuously stirring and then left the flow of fluid for a little while. Bodewadt flow is additionally perceived further in many procedures such as hurricanes, food-processing industries, tornadoes, turbo-machinery and chemical mixing chambers etc. Rafiq et al.^12^ examined numerical assessment of nanofluid for Bodewadt slip flow above a convectively heated penetrable disk. Sahoo et al.^13^ obtained numerical solution of viscous fluid for steady Bodewadt flow using finite difference and keller-box method. Muhammad et al.^14^ formulated entropy production in three-dimensional Bodewadt flow of nanofluid over an infinite stationary disk. Mukherjee and Sahoo^15^ discussed the impact of radial stretch of the lower disk on the boundary-layer Bodewadt flow in the existence of the Coriolis force. Hina et al.^16^ gives the numerical solution of rotating Bodewadt flow above a porous disk of micropolar fluid. Some recent attempts related to Bodewadt flow can be seen in Refs.^17–22^.

Heat transfer is the field of thermal engineering which deals with the exchange, conversion, generation and use of thermal energy among physical systems (i.e.: heat energy). Transfer of heat is categorized within various mechanisms such as radiation, convection and conduction. Transfer of heat has broad range of applications in climate engineering, architecture, industries of chemical process, greenhouse effect and heat transfer in the body of human beings. There is an immense impact of temperature gradients on characteristics of fluid. Particularly, there is a direct relationship between temperature and viscosity in gases while an inverse relationship between temperature and viscosity is in liquids. Variable characteristics of fluid flows are often perceived in applications associated with aerodynamics & aero-acoustics. Takhar et al.^23^ examined the impacts of fluid properties that depends upon temperature on boundary layer flow above a continuous moving surface. They considered two cases, namely, variable fluid viscosity and constant fluid properties. Salahuddin et al.^24^ examined the rotating behavior of 2nd -grade fluid with mass & heat transfer effects between two parallel plates. Rafiq et al.^25^ formulated the impacts of heat transport in fluid flow produced by a revolving permeable disk with variable characteristics of fluid. Ahmed et al.^26^ numerically modeled the unsteady MHD flow and heat transfer with variable viscosity of nanofluid in carbon nanotubes over a shrinking porous surface. Adnan et al.^27^ analyzed numerically the heat transfer in the nanofluid composed by silver nanomaterials and nanodimaond. Several attempts in this regime has been made by investigators includes^28–30^.

The heat which is produced due to the flow of an electric current through a conductor is called joule heating, also known as ohmic heating. Joule heating have a vast range of applications in our daily life like as glowing of filament of an incandescent light bulb, electrical fuses, hotplate of electrical tabletop etc. Viscous dissipation is an irreversible procedure in which the work is done by a fluid on adjoining layers owing to the action of shear forces is converted into heat. Repercussions of viscous dissipation plays a crucial role in natural convection in numerous devices that are subjected to big variations of gravitational force or which operate at high speed. Jawad et al.^31^ discussed the analysis of transfer of heat and entropy generation in MHD flow of carbon nanotubes with the impacts of viscous dissipation and thermal radiation. Afridi et al.^32^ described the chaos analysis in stagnation point flow with MHD, ohmic heating & fluid friction impact. Entropy analysis in MHD nanofluid due to a heated stretching sheet with viscous dissipation & radiation are analyzed by Sithole et al.^33^. Zubair et al.^34^ investigated the entropy generation optimization in three-dimensional MHD flow between two parallel rotating plates of casson nanofluid with viscous dissipation & ohmic heating effects. Lund et al.^35^ gives the numerical solution of MHD flow of micro polar fluid under the impacts of joule heating and viscous dissipation over a shrinking sheet. Rasheed et al.^36^ examined the MHD flow of chemically reactive casson liquid with effects of viscous dissipation and heat source over a penetrable stretching surface. The latest investigations in this regime include studies^37–40^.

Fluids play dominant role in the application of flow rate and heat transfer in numerous engineering systems, biomedical procedures and technological developments. Gases and liquids are known as fluids because they can be made to move, or flow. The molecules of any fluid themselves are in constant, random movement, colliding with each other and with the walls of the container. A fluid is said to be viscous or real fluid if it doesn’t flow easily because these fluids have finite values of viscosity that apply the shearing stress force on a surface with which is during a contact. These fluids have more resistance to flow. These fluids have very broad range of applications in our practical life. Equipment used for manufacturing requires suitable lubrication to run smoothly. The oil used as a lubricant for parts of heavy machinery should have a high viscous coefficient. Circulation of blood via veins and arteries rely on the viscosity of fluids. Few of the viscous fluids add texture to foods: for example, honey is quite viscous and can convert the “mouth feel” of a dish. The highly viscous liquid is utilized to damp the movement of some instruments and is used as brake oil in hydraulic brakes. Ayub et al.^41^ analyzed numerically the free convection flow of MHD viscous fluid with heat generation and Newtonian heating over a rotating vertical plate. Yong-Min Li et al.^42^ explained the mathematical modeling of entropy optimization due to a rotating cone in a convective viscous fluid flow.

Magnetohydrodynamics describe the study of movement of electrically conducting fluid (e.g. liquid metals and plasmas) in the presence of a magnetic field. The key hypothesis behind magnetohydrodynamics is that magnetic fields can generate current in a moving conductive fluid, which sequentially produce a force on the fluid and also alter the magnetic field itself. MHD have broad variety of applications in the field of Astrophysics, Engineering, Geophysics and Magnetic drug targeting. Shah et al.^43^ analyzed the radiative effect radiation in magnetohydrodynamic casson nanofluid flow along with entropy generation and chemical reactions above a nonlinearily stretching sheet. Awais et al.^44^ explained the mathematical modeling of prandtl MHD melted fluid flow towards an inclined cylindrical surface. Khan et al.^45^ explained the entropy optimization rate in magnetohydrodynamic flow of viscous liquids with chemical reactions due to curved stretchable surface. Alreshidi et al.^46^ discussed the thermophoresis effects and brownian movement in an incompressible and time independent flow of MHD nanofluid due to permeable revolving disk. Bibi et al.^47^ presented theoretical analysis of magnetohydrodynamic carreau fluid above a revolving disk with the aid of von-karman transformations. Some remarkable applications regarding magnetohydrodynamics are cited here^48–52^.

By utilizing the knowledge of pre- mentioned literature, the objectives of present research is:

- To find numerical solution of heat transfer effects in a Bodewadt flow in existence of joule heating, viscous dissipation, magnetic field and internal heat generation/ absorption.
- Kinetics of viscous fluid with variable characteristics of fluid having solid body rotation above a permeable disk (having cylindrical geometry) are analyzed.
- Utilizing the usual Von-Karman variables to convert the governing equations of heat transfer (with variable fluid properties) & fluid motion into self-similar differential equations which are then analyzed numerically by using Adams Bashforth method.
- Results are manifested to scrutinize the behavior of velocity and temperature profiles for different emerging parameters with the assistance of graphs, tables & bar charts.
- Outcomes of current investigations are compared with prior work, to ensure the authenticity of the numerical method, and strong agreement is noted.

## Problem development and governing model

**Figure 1:**
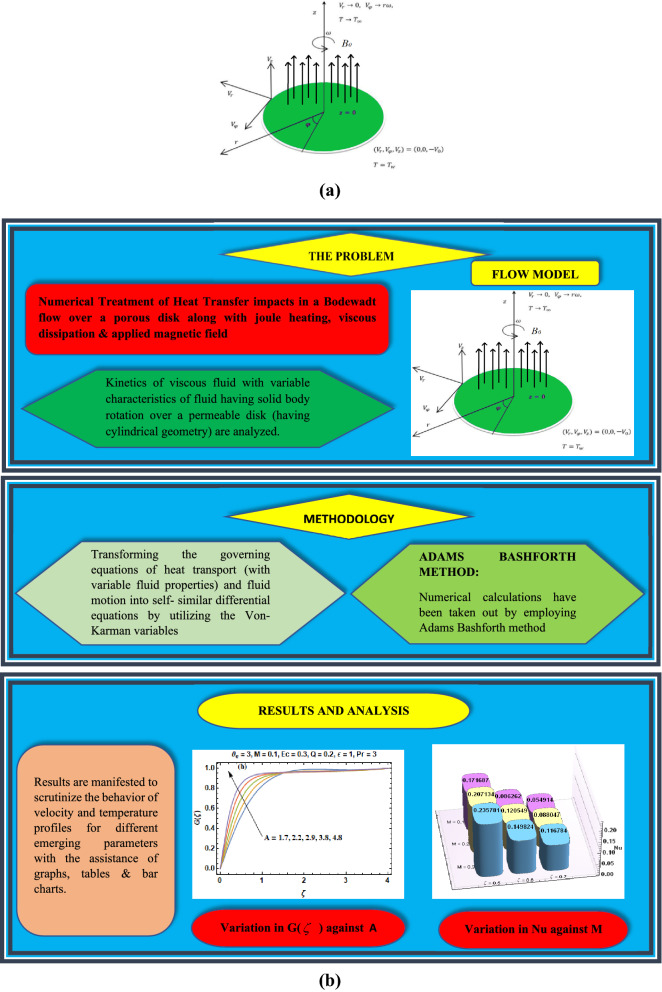
(**a)** Physical configuration and coordinate system. (**b**) Process flow architecture.

Let us assume the flow of viscous fluid with variable characteristics of fluid is in the condition of rigid body rotation over a penetrable disk through its origin as portrayed in the Fig. 1a, whereas the complete flow chart architecture is depicted via Fig. 1b. The disk rotates around vertical axis with uniform angular velocity $\omega$. The components of velocity $\left( {V_{r} , \, V_{\varphi } , \, V_{z} } \right)$ are taken in the direction of cylindrical coordinates $\left( {r, \, \phi , \, z} \right)$. The disk temperature is supposed constant on $T_{w}$ while the temperature of exterior flow is considered as $T_{\infty }$. A uniform magnetic field of strength $B_{0}$ is functional along the z-direction while the induced magnetic field is presumed to be negligible.

The equation of motion, Navier- Stoke’s equation and thermal energy equation with heat generation and absorption, ohmic heating & viscous dissipation takes the form as mentioned below:

1 $$\frac{{\partial (rV_{r} )}}{\partial r} + \frac{{\partial (rV_{z} )}}{\partial z}{ = 0,}$$

2 $$\rho \left( {V_{r} \frac{{\partial V_{r} }}{\partial r} + V_{z} \frac{{\partial V_{r} }}{\partial z} - \frac{{V_{\varphi }^{2} }}{r}} \right) + \sigma B_{0}^{2} V_{r} = - \frac{\partial p}{{\partial r}} + \frac{{\partial \tau_{rr} }}{\partial r} + \frac{{\partial \tau_{rz} }}{\partial z} + \frac{{\tau_{rr} - \tau_{\varphi \varphi } }}{r},$$

3 $$\rho \left( {V_{r} \frac{{\partial V_{\varphi } }}{\partial r} + V_{z} \frac{{\partial V_{\varphi } }}{\partial z} + \frac{{V_{r} V_{\varphi } }}{r}} \right) + \sigma B_{0}^{2} V_{\varphi } = \frac{1}{{r^{2} }}\frac{\partial }{\partial r}\left( {r^{2} \tau_{r\varphi } } \right) + \frac{\partial }{\partial z}\tau_{z\varphi } + \frac{{\tau_{r\varphi } - \tau_{\varphi r} }}{r},$$

4 $$\rho \left( {V_{r} \frac{{\partial V_{z} }}{\partial r} + V_{z} \frac{{\partial V_{z} }}{\partial z}} \right) = \frac{1}{r}\frac{\partial }{\partial r}\left( {r\tau_{rz} } \right) + \frac{\partial }{\partial z}\tau_{zz}$$

5 $$\begin{gathered} \rho c_{P} \left( {V_{r} \frac{\partial T}{{\partial r}} + V_{z} \frac{\partial T}{{\partial z}}} \right) = - \nabla .\left[ { - k\left( T \right).\nabla T} \right] + \sigma B_{0}^{2} \left( {V_{r}^{2} + V_{\varphi }^{2} } \right) + Q^{*} \left( {T - T_{\infty } } \right) \hfill \\ \, + \mu \left[ {2\left\{ {\left( {\frac{{\partial V_{r} }}{\partial r}} \right)^{2} + \left( {\frac{{V_{r} }}{r}} \right)^{2} + \left( {\frac{{\partial V_{z} }}{\partial z}} \right)^{2} } \right\} + \left( {\frac{{\partial V_{\varphi } }}{\partial z}} \right)^{2} + \left( {\frac{{\partial V_{r} }}{\partial z} + \frac{{\partial V_{z} }}{\partial r}} \right)^{2} + \left( {r\frac{\partial }{\partial r}\left( {\frac{{V_{\varphi } }}{r}} \right)} \right)^{2} } \right], \hfill \\ \end{gathered}$$

Here $\left( {V_{r} , \, V_{\phi } {\text{ and }}V_{z} } \right)$ in the above equations represents the constituents of fluid velocity in the radial, tangential and axial directions, $\left( \rho \right)$ indicates the fluid density, $\left( {C_{p} } \right)$ indicates the specific heat capacity, $\left( {B_{0} } \right)$ represents the magnetic field intensity, $k(T)$ stands for thermal conductivity, $\left( \mu \right)$ indicates the dynamic viscosity, $\left( T \right)$ denotes the temperature of fluid, $\left( \sigma \right)$ represents the electrical conductivity, $\left( {T_{\infty } } \right)$ represents the free stream temperature and $\left( {Q^{*} } \right)$ stands for coefficient of heat generation/absorption . The stress tensor components are given as:

6 $$\begin{gathered} \tau_{rr} = \, \mu \left( {2\frac{{\partial V_{r} }}{\partial r}} \right), \, \tau_{\varphi \varphi } = \, \mu \left( {\frac{{2V_{r} }}{r}} \right), \, \tau_{zz} = \, \mu \left( {2\frac{{\partial V_{z} }}{\partial z}} \right), \hfill \\ \tau_{\varphi z} = \, \tau_{z\varphi } = \, \mu \left( {\frac{{\partial V_{\varphi } }}{\partial z}} \right), \, \tau_{rz} = \, \tau_{zr} = \, \mu \left( {\frac{{\partial V_{r} }}{\partial z} + \frac{{\partial V_{z} }}{\partial r}} \right), \, \tau_{r\varphi } = \, \tau_{\varphi r} = \, \mu \left( {\frac{{\partial V_{\varphi } }}{\partial r} - \frac{{V_{\varphi } }}{r}} \right), \, \hfill \\ \end{gathered}$$

We presume that viscosity $\left( \mu \right)$ manifests an inverse linear dependency of temperature.

7 $$\mu = \frac{{\mu_{\infty } }}{{1 + \gamma (T - T_{\infty } )}},$$

Here $\left( {\mu_{\infty } } \right)$ represents ambient viscosity of the fluid and $\gamma$ denotes a constant. When we increase the temperature, the viscosity of the liquids decreases $\left( {\gamma < 0} \right)$ whereas the viscosity of air and other gases increases $\left( {\gamma > 0} \right)$. Equation (7) may also be rewritten in the form as given below:

$$\frac{1}{\mu } = \alpha \left( {T - T_{e} } \right)$$ where $$T_{e} = T_{\infty } - \frac{1}{\gamma }$$ and $$\alpha = \frac{\gamma }{{\mu_{\infty } }}$$.

We presumed thermal conductivity that is dependent on the temperature is of the shape $k(T) = k_{\infty } \left( {1 + \frac{{\varepsilon \left( {T - T_{\infty } } \right)}}{{\left( {T_{w} - T_{\infty } } \right)}}} \right)$, in which $K_{\infty }$ represents thermal conductivity at ambient. The suitable boundary conditions for the considered flow model are defined as:

8 $$\begin{gathered} At \, z = 0: \, V_{r} = 0, \, V_{\varphi } = 0, \, V_{z} = - V_{0} , \, T = T_{w} , \hfill \\ as \, z \to \infty : \, V_{r} \to 0, \, V_{\varphi } \to r\omega , \, T \to T_{\infty } . \hfill \\ \end{gathered}$$

Note that the centrifugal force is well balanced by the radial pressure gradient in the exterior flow. Mathematically, we can write it as:

9 $$\frac{\partial p}{{\partial r}} = \rho r\omega^{2}$$

### Similarity transformation

Depending upon the scale of length $$\sqrt {{\raise0.7ex\hbox{${v_{\infty } }$} \!\mathord{\left/ {\vphantom {{v_{\infty } } \omega }}\right.\kern-\nulldelimiterspace} \!\lower0.7ex\hbox{$\omega $}}}$$, we write the similarity variable (too called dimensionless distance) as $$\zeta = z\left( {{\raise0.7ex\hbox{${\nu_{\infty } }$} \!\mathord{\left/ {\vphantom {{\nu_{\infty } } \omega }}\right.\kern-\nulldelimiterspace} \!\lower0.7ex\hbox{$\omega $}}} \right)^{{ - \, \frac{1}{2}}}$$. As the scale of velocity is $$\sqrt {v_{\infty } \omega }$$ and scale of time is $$\omega^{ - 1}$$, therefore, we introduce the dimensionless constituents of velocity (F, G, H) & profile of temperature $$\theta$$ without dimensions as follows:

10 $$F\left( \zeta \right) = \frac{{V_{r} }}{r\omega }, \, G\left( \zeta \right) = \frac{{V_{\varphi } }}{r\omega }, \, H(\zeta ) = \frac{{V_{z} }}{{\sqrt {v_{\infty } \omega } }}, \, \theta \left( \zeta \right) = \frac{{T - T_{\infty } }}{{T_{w} - T_{\infty } }}$$

By applying boundary layer approximations and then using formulas (10) in Eqs. (1–5); we acquire:

11 $$2F + H^{\prime} = 0,$$

12 $$F^{\prime\prime} - \frac{1}{{\theta - \theta_{e} }}F^{\prime}\theta ^{\prime} + \frac{{\theta - \theta_{e} }}{{\theta_{e} }}MF + \frac{{\theta - \theta_{e} }}{{\theta_{e} }}\left( {F^{2} + HF^{\prime} - G^{2} + 1} \right) = 0,$$

13 $$G^{\prime\prime} - \frac{1}{{\theta - \theta_{e} }}G^{\prime}\theta ^{\prime} + \frac{{\theta - \theta_{e} }}{{\theta_{e} }}MG + \frac{{\theta - \theta_{e} }}{{\theta_{e} }}\left( {2FG + HG^{\prime}} \right) = 0,$$

14 $$H\theta ^{\prime} - \left( {\frac{1}{\Pr }} \right)\left[ {\left( {1 + \varepsilon \theta } \right)\theta ^{\prime\prime} + \varepsilon \theta ^{{\prime}{2}} } \right] - MEc(F^{2} + G^{2} ) - Q\theta + \left( {\frac{{\theta_{e} }}{{\theta - \theta_{e} }}} \right)Ec[F^{{\prime}{2}} + G^{{\prime}{2}} ] = 0,$$

The boundary conditions are converted into:

15 $$\begin{gathered} H\left( \zeta \right) = - A, \, F(\zeta ) = 0, \, G(\zeta ) = 0, \, \theta (\zeta ) = 1,as \, \zeta \to 0 \hfill \\ F(\zeta ) \to 0, \, G(\zeta ) \to 1, \, \theta (\zeta ) \to 0 \, as \, \zeta \to \infty . \hfill \\ \end{gathered}$$

In the above mentioned expressions (11–15), $A = \frac{{V_{0} }}{{\sqrt {v_{\infty } \omega } }}$ denotes the wall suction parameter, $\Pr = \frac{{\mu_{\infty } c_{p} }}{{k_{\infty } }}$ denotes the value of Prandtl number at the ambient, $Ec = \frac{{r^{2} \omega^{2} }}{{c_{p} (T_{w} - T_{\infty } )}}$ is the Eckert number, $M = \frac{{\sigma B_{0}^{2} }}{\rho \omega }$ denotes the magnetic field parameter, $\theta_{e} = \frac{{T_{e} - T_{\infty } }}{{T_{w} - T_{\infty } }} = \frac{ - 1}{{\gamma \left( {T_{w} - T_{\infty } } \right)}}$ indicates the dimensional constant and $Q = \frac{{Q^{*} }}{{\rho c_{P} \omega }}$ represents the parameter of heat generation/absorption.

### Nusselt number and Skin friction component:

We define skin friction parameter as given below:

16 $$C_{f} = \frac{{\sqrt {\tau_{r}^{2} + \tau_{\varphi }^{2} } }}{{\rho (r\omega )^{2} }}$$

where $\tau_{\varphi }$ denotes the circumferential wall stress and $\tau_{r}$ represents radial wall stress that can be evaluated by utilizing the Newtonian formulas as:

17 $$\tau_{r} = \mu \left( {\frac{{\partial V_{r} }}{\partial z} + \frac{{\partial V_{z} }}{\partial r}} \right)_{z = 0} = r\omega \frac{{\mu_{\infty } }}{{\gamma (T_{w} - T_{\infty } )\theta (0) + 1}}\sqrt {\frac{\omega }{{v_{\infty } }}} F^{\prime}(0)$$

18 $$\tau_{\varphi } = \mu \left( {\frac{{\partial V_{\varphi } }}{\partial z} + \frac{{\partial V_{z} }}{\partial r}} \right)_{z = 0} = r\omega \frac{{\mu_{\infty } }}{{\gamma (T_{w} - T_{\infty } )\theta (0) + 1}}\sqrt {\frac{\omega }{{v_{\infty } }}} G^{\prime}(0)$$

By utilizing expressions (7 and 10) in expressions (16, 17 & 18), we obtain:

19 $$C_{f} = {\text{Re}}_{r}^{{ - \frac{1}{2}}} \frac{{\theta_{e} }}{{\theta_{e} - \theta }}\sqrt {F^{\prime}(0)^{2} + G^{\prime}(0)^{2} } .$$

Here ${\text{Re}}_{r} = \frac{{r\omega^{2} }}{{v_{\infty } }}$ indicates the rotational Reynolds number. Additionally, we define the local nusselt number as follows:

20 $$Nu = \frac{{Lq_{w} }}{{k\left( {T_{w} - T_{\infty } } \right)}}$$

in which $L = \sqrt {\frac{{v_{\infty } }}{\omega }}$ denotes the length scale &$q_{w} = - k\left( {\frac{\partial T}{{\partial z}}} \right)_{z = 0}$ represents the wall heat flux of the current research problem. By using formulas (10) in expression (20), we get:

21 $$Nu = - \, \theta ^{\prime}(0).$$

## Numerical consequences and discussion:

**Figure 2:**
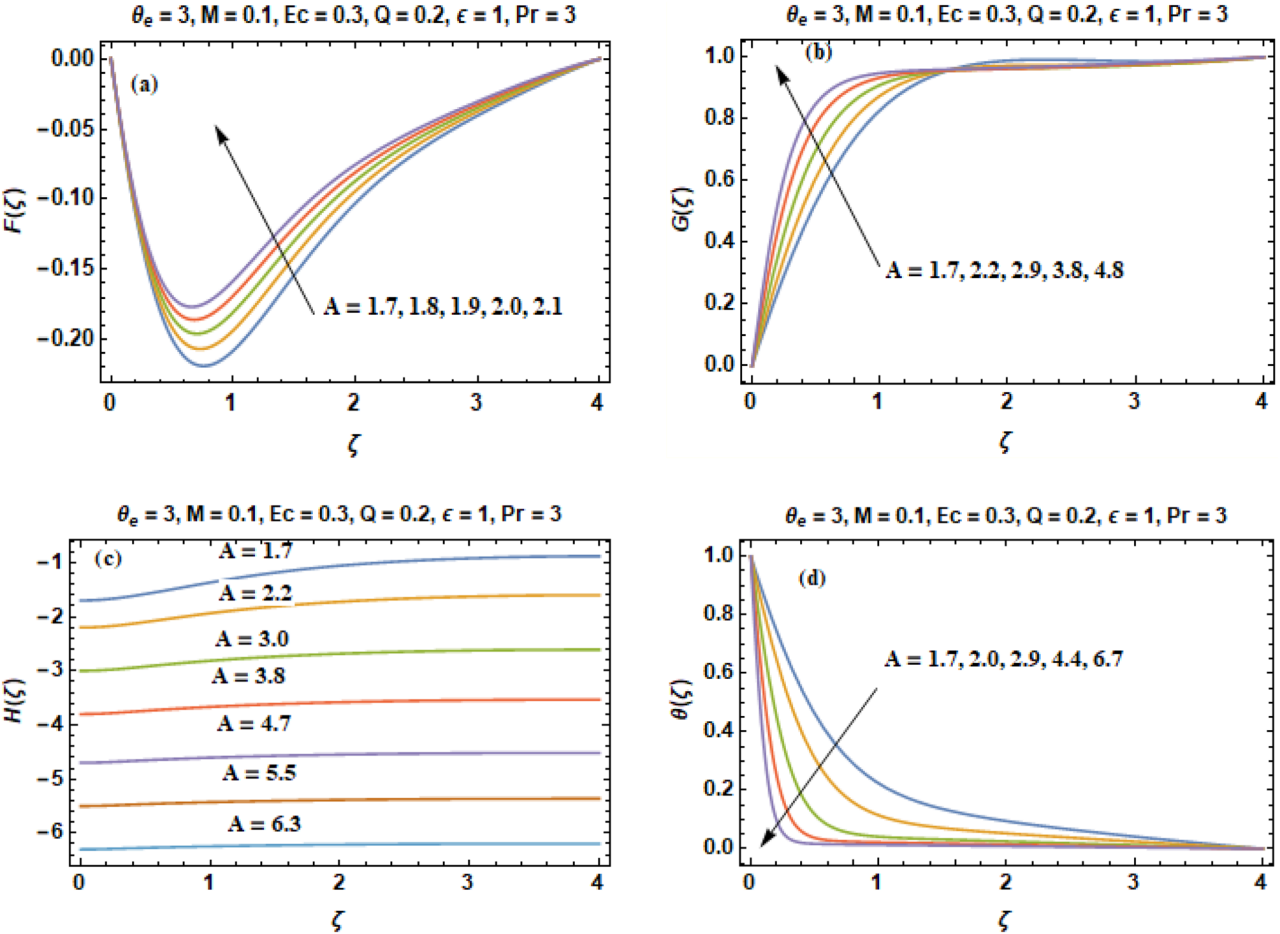
(**a**–**d**) Components of velocity (**F**, **G**, **H**) & temperature curves (**θ**) versus **A**.

In this portion, heat transfer influences in a Bodewadt flow with joule heating, viscous dissipation, magnetic field and internal heat generation and absorption that are evaluated by taking variable properties of fluid are discussed. Using Adams Bashforth method, numerical computations are obtained for velocity constituents & temperature profile for distinct values of the concerned parameters in the range $$0.2 \le \Pr \le 5.0,$$$$1.7 \le A \le 6.7,$$$$0.1 \le M \le 1.2,$$$${ - 100} \le \theta_{e} \le 100,$$$${ - }0.9 \le Q \le 3.6,$$$$0 \le Ec \le 1.2,$$$${0} \le \varepsilon \le 4.1.$$ The graphical illustration of velocity constituents (F, G, H) & temperature profile ($$\theta$$) for a variety of suction strength parameter are illustrated in Fig. 2a-d. The direction of inward flow of radial velocity F as portrayed in Fig. 2a is diminished monotonically with an increment in the value of A and this is escorted with a reduction of axial flow H as shown in Fig. 2c. However, the radial inward flow generated by the pressure gradient decelerates with an enhancement in the value of wall suction parameter A. The impact of suction leads to betterment in the frigidity rate of wall at a vast scale that is beneficial in numerous technical processes. The direction of the axial flow is directed upward in the absenteeism of wall suction, no matter how much greater the quantity of roughness parameter may be. The circumferential component of velocity G remains constant away from the disk while it is decreased significantly close to the disk due to the viscous drag as portrayed in Fig. 2b.

The axially independent radial pressure gradient compels the fluid particles close to the disk to move in the direction of rotational axis. For that reason, the direction of the axial flow in the Bodewadt flow is directed upwards as seen in Fig. 2c. The axially upward flow results into the non-physical solution of heat energy equation. Figure 2c illustrates that axial component of velocity becomes constant when we applied an appropriate value of suction velocity. All the three constituents of velocity manifest oscillations whose amplitude shrinks when axial distance increases. When A ≥ 1, the direction of the axial flow is inverted such that the fluid instead moves towards the solid surface. Depth of heat penetration also decreases when suction velocity increases. The results of Fig. 2c depicts that when larger A is accounted than greater quantity of (cold) fluid is transferred in the direction of the disk. As a result, layer of thermal boundary is suppressed with an increment in the velocity of wall suction and boosts transfer of heat from the disk as portrayed in Fig. 2d. Lastly, the axially upward flow is a consequence of an unphysical solution to the problem of advection–diffusion. To overcome this physical incompatibility, we can reverse the direction of the axial flow through the application of sufficiently high suction velocity. Therefore, it can be stated that the effect of damping arising as a result of wall suction can overcome the absolute or convective type instabilities in Bodewadt flow known from the published literature.

**Figure 3:**
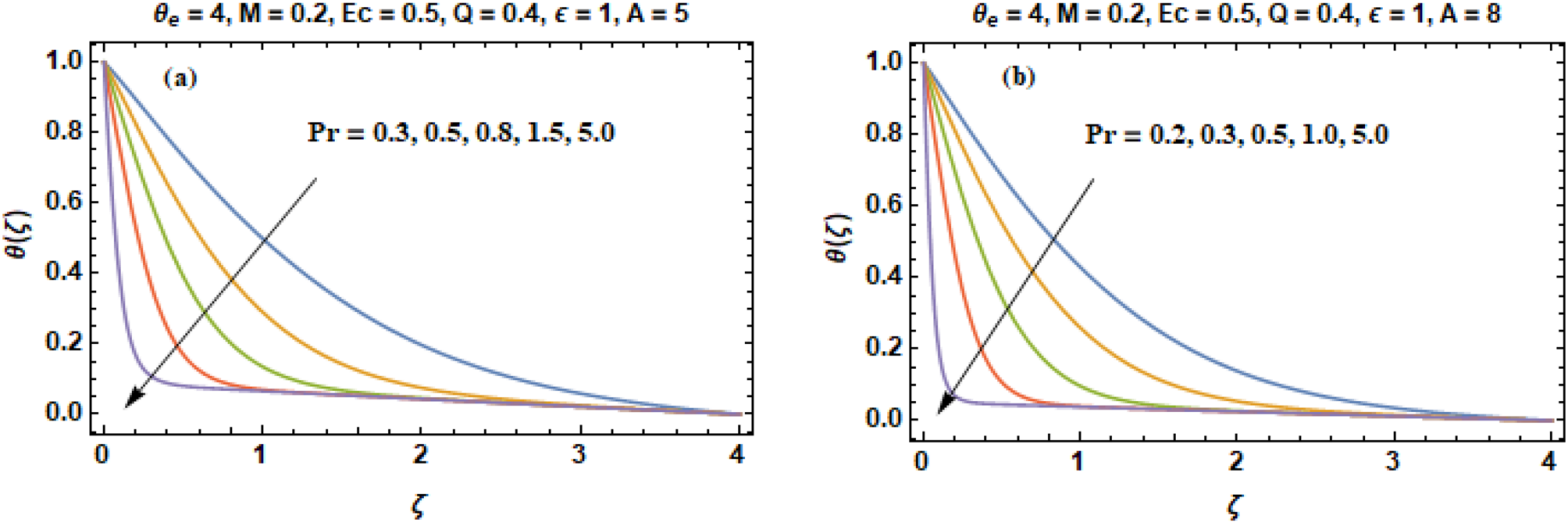
(**a**, **b**) Temperature curves **(θ)** versus **Pr** when (**a**)** A = 5** and (**b**)** A = 8**.

Figure 3a, b includes the curves of temperature (θ) obtained by changing the value of prandtl number in two cases (A = 5 & A = 8). It is suggested from the comparison of Fig. 3a, b that when velocity of wall suction increases than the deepness of heat penetration is much suppressed. It can be noted instantly that an enhancement in the values of prandtl number rises the temperature of the fluid in the vicinity of the disk. The graphs demonstrate that the thickness of layer of thermal boundary and fluid temperature decreases with an increment in the prandtl number on a fixed value of $\zeta$. The reason behind this is that prandtl number (Pr) is termed as fraction of momentum diffusion to thermal diffusion. When thermal diffusivity reduces, automatically the value of prandtl number (Pr) increases and fluid temperature decreases. In other words, temperature and prandtl number have an inverse relationship. The Prandtl number is a non-dimensional number, defined as the ratio of momentum diffusivity to thermal diffusivity. Large values of the prandtl number (Pr >  > 1) means the momentum diffusivity dominates the behavior while with small values of the prandtl number (Pr <  < 1), the thermal diffusivity dominates the behavior. In heat transfer problems, the prandtl number controls the relative thickness of the thermal and momentum boundary layers. The prandtl numbers of gases are approximately equal to 1, which denotes that both heat and momentum dissipate through the fluid at about the same rate. Heat diffuses very slowly in oils (Pr >  > 1) and very quickly in liquid metals (Pr <  < 1) relative to momentum. As a result layer of thermal boundary is much thinner for oils and much thicker for liquid metals relative to velocity boundary layer. However, the quantity of $\theta ^{\prime}\left( 0 \right)$ increases considerably (in absolute sense) signaling a betterment in the rate of heat transition from the disk.

**Figure 4:**
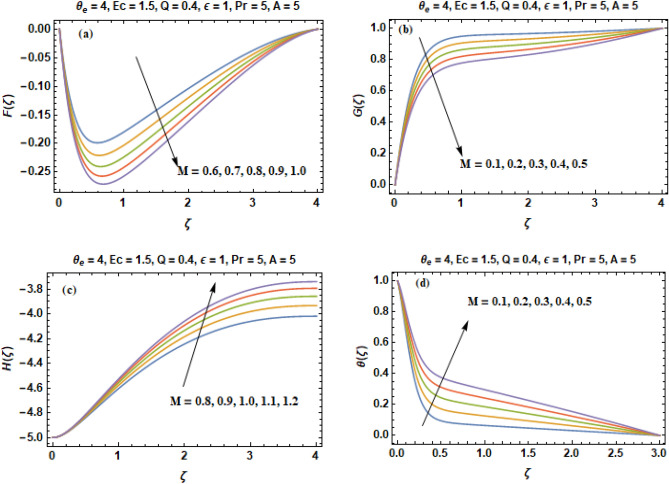
(**a–d**) Constituents of velocity **(F, G, H)** & temperature profile **(θ)** versus Magnetic field parameter **M**.

The influences of magnetic interaction parameter (M) on the velocity constituents (F, G, H) in $\left( {r, \, \phi , \, z} \right)$ directions and temperature curves ($\theta$) can be visualized in Fig. 4a–d. The radial, circumferential and axial components of velocity are decreasing gradually with intensification of strength of magnetic field (M) as evinced in Fig. 4a–c. The physics behind this is that an increase in the magnetic parameter (M) has the impact of damping the velocity profiles of fluid. This is because the direction of flow that is normal to the transverse magnetic field will result in a resistive force named as Lorentz force that is identical to the drag force which resist the fluid flow and as a consequence reduces the radial (F), axial (H) and tangential (G) velocities. Behavior of temperature curves (θ) for non- identical values of magnetic field parameter M is illustrated in Fig. 4d. Contrary to the profiles of velocity, temperature (θ) enhances when we increase the value of M. This is due to the fact that bigger drag co-efficient on the surface owing to sturdy magnetic field offers resistance to the particles of fluid. As a consequence produces heat owing to which temperature (θ) enhances. In modern technology, magnetic fields are especially used in electro mechanics & electrical engineering. Magnetic fields of rotating behavior are utilized in both electric generators and electric motors. Magnetic forces provide information about the charge carriers in a material via Hall effect. In electrical devices such as transformers, the magnetic field interaction is conceptualized and scrutinized as magnetic circuits. The Earth creates its own magnetic field, which protects the ozone layer of Earth from the solar wind and is significant in navigation using a compass.

**Figure 5:**
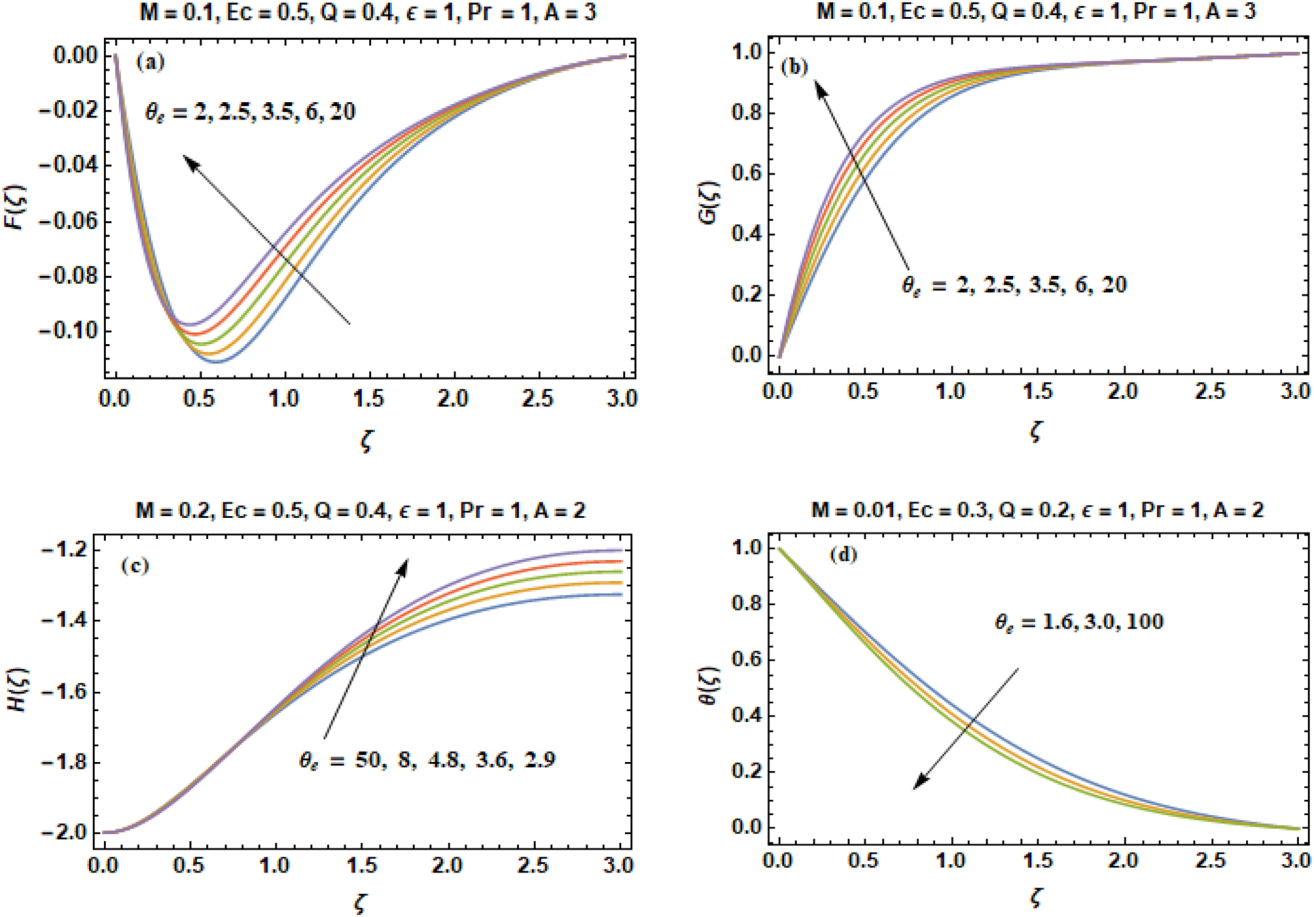
(**a–d**) Components of velocity **(F, G, H)** & temperature profile **(θ)** versus variable viscosity parameter $\theta_{e}$ > 0.

Figure 5a–d illustrates the change in velocity constituents (F, G, H) & temperature curves (θ) by altering the value of variable viscosity parameter ($$\theta_{e}$$ > 0). In these figures, the quantity of prandtl number Pr is kept persistent at Pr = 1 that is an adequate selection for air & numerous more gases. The variable viscosity parameter ($$\theta_{e}$$) elasticities the significance of the quantity $${\raise0.7ex\hbox{$1$} \!\mathord{\left/ {\vphantom {1 \gamma }}\right.\kern-\nulldelimiterspace} \!\lower0.7ex\hbox{$\gamma $}}$$ which is in direct proportion to the rate of dependence of viscosity on temperature of the fluid. Viscosity is a physical property that explores fluid’s internal resistance towards its motion and oppose the shear deformation rate. A fluid having low viscosity easily flows because its molecular makeup results in very small amount of friction when it is in movement. A fluid with large viscosity resists motion because its molecular makeup gives it a lot of internal friction. We suppose the viscosity of fluid to vary as an exponential function of temperature in the dimensionless form, whereas the constant value of viscosity coefficient far away from the disk, is the variable viscosity parameter. When ($$\theta_{e} \to \infty$$), the fluid viscosity becomes equivalent to the ambient viscosity whereas the viscosity of fluid becomes a sturdy function of temperature when $$\theta_{e} \to 0$$. Figure 5c depicts that the axial velocity expedites in the downward direction as $$\theta_{e}$$ increases. As a consequence, this effect causes a reduction in the severity of the radial flow as illustrated in Fig. 5a. The value of entrainment velocity H (∞) is also increased indicating a growth in the fluid volume that is sucked in the direction of the disk. The circumferential component of velocity G accelerated in the upward direction when the value of $$\theta_{e}$$ increases as shown in Fig. 5b. As a result, the depth of heat penetration is (slightly) diminished and rate of heat transition is augmented when $$\theta_{e}$$ is increased as portrayed in Fig. 5d.

**Figure 6:**
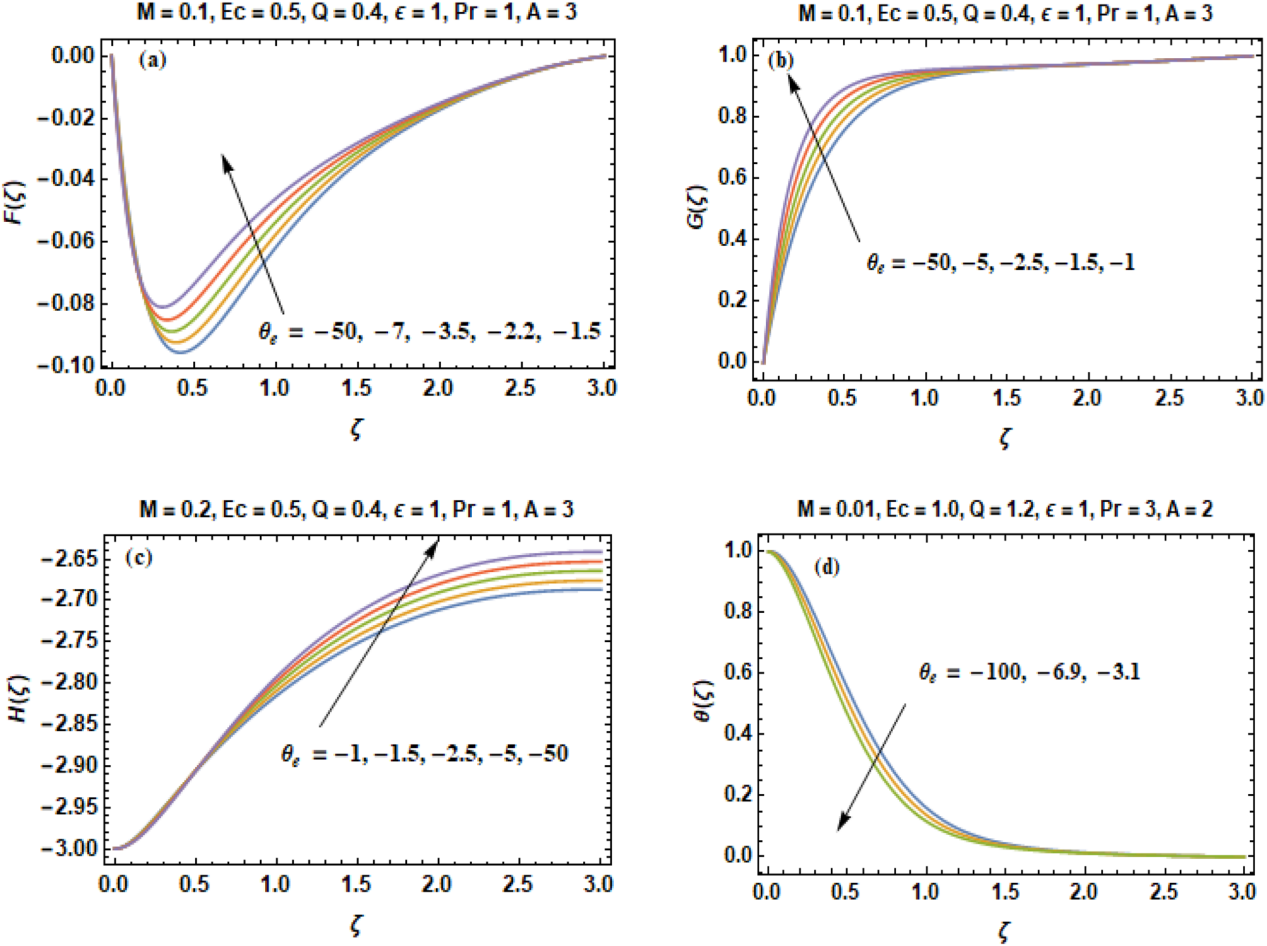
(**a–d**) Components of velocity **(F, G, H)** & temperature profile **(θ)** versus variable viscosity parameter $\theta_{e}$ < 0.

In Fig. 6a–d, we calculate velocity constituents and temperature curves for various values of variable viscosity parameter $\theta_{e}$ when ($\theta_{e}$ < 0). In such situation, the viscosity of the fluid has an inverse relationship with temperature. Different to the outcomes of Fig. 5c, the axial component of velocity decreases when we increase the value of $\theta_{e}$ that in results to the thickening of layer of thermal boundary as illustrated in Fig. 6d. Figure 6b reveals that the tangential velocity accelerates in the upward direction when the value of $\theta_{e}$ increases.

**Figure7:**
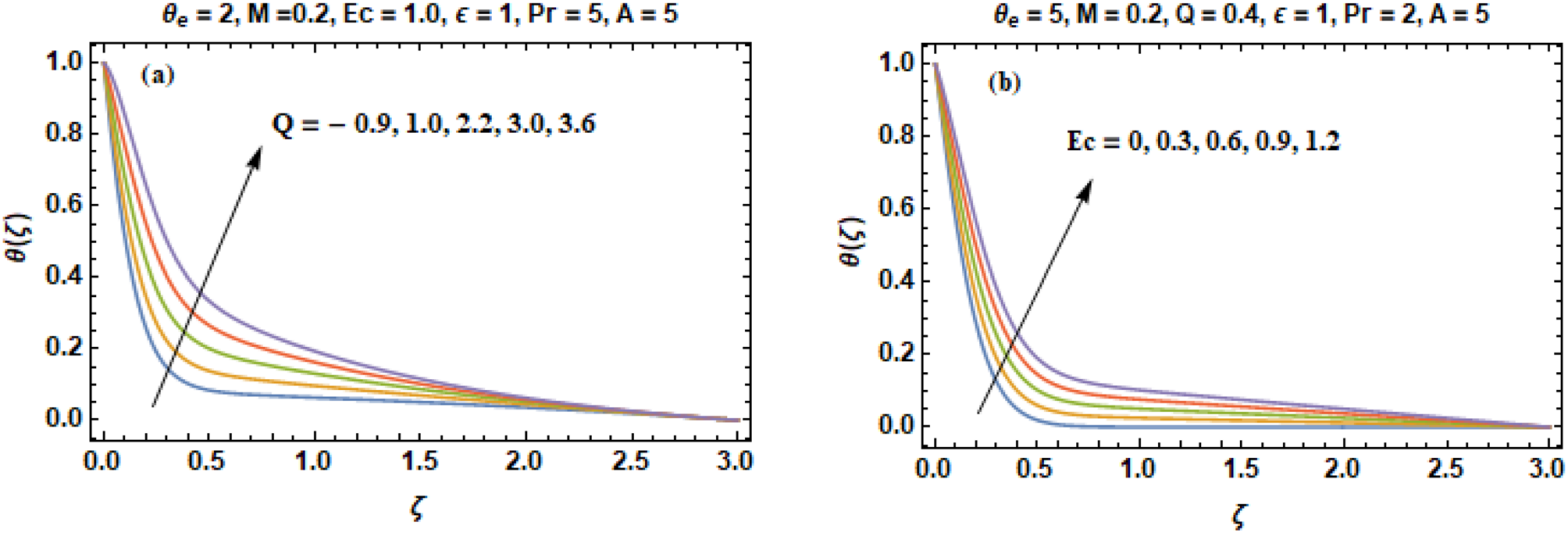
(**a–b**) Temperature curves **(θ)** versus heat generation/absorption **Q** and Eckert number **Ec**.

The influences of heat generation/absorption (Q) and Eckert number (Ec) are demonstrated in Fig. 7a–b. In Fig. 7a, negative values of parameter (Q) are representing the heat absorption (Q < 0) and positive values are corresponding to heat generation (Q > 0). It is obvious from the Fig. 7a that an enhancement in the heat generation produces an increase in the thickness of thermal boundary layer whereas an increment in the heat absorption yields a decrease in the thickness of thermal boundary layer. An increment in the absorption of heat implies that the heat is withdrawn by some means from the system. That is the reason that why an enhancement in the parameter of heat absorption results a reduction in the thickness of layer of thermal boundary and an increment in the rate of heat transport. Contrary to this, an increment in the positive values manifest strong heat generation that enhances the fluid temperature in accordance with the definition of heat generation. Figure 7b portrays the repercussions of Eckert number on the temperature profile. An increase in the temperature is observed for greater values of Ec. An Eckert number (Ec) is utilize to calculate the loss of energy during configuration of flow. It tells us the relationship between enthalpy difference and kinetic energy of liquid particles, and is used to characterize heat transfer dissipation. For an augmentation in the values of Eckert number from 0 to 1.2, the mechanical energy of the fluid is transformed into thermal energy because of internal friction of molecules. Hence the temperature of the liquid increases.

**Figure 8:**
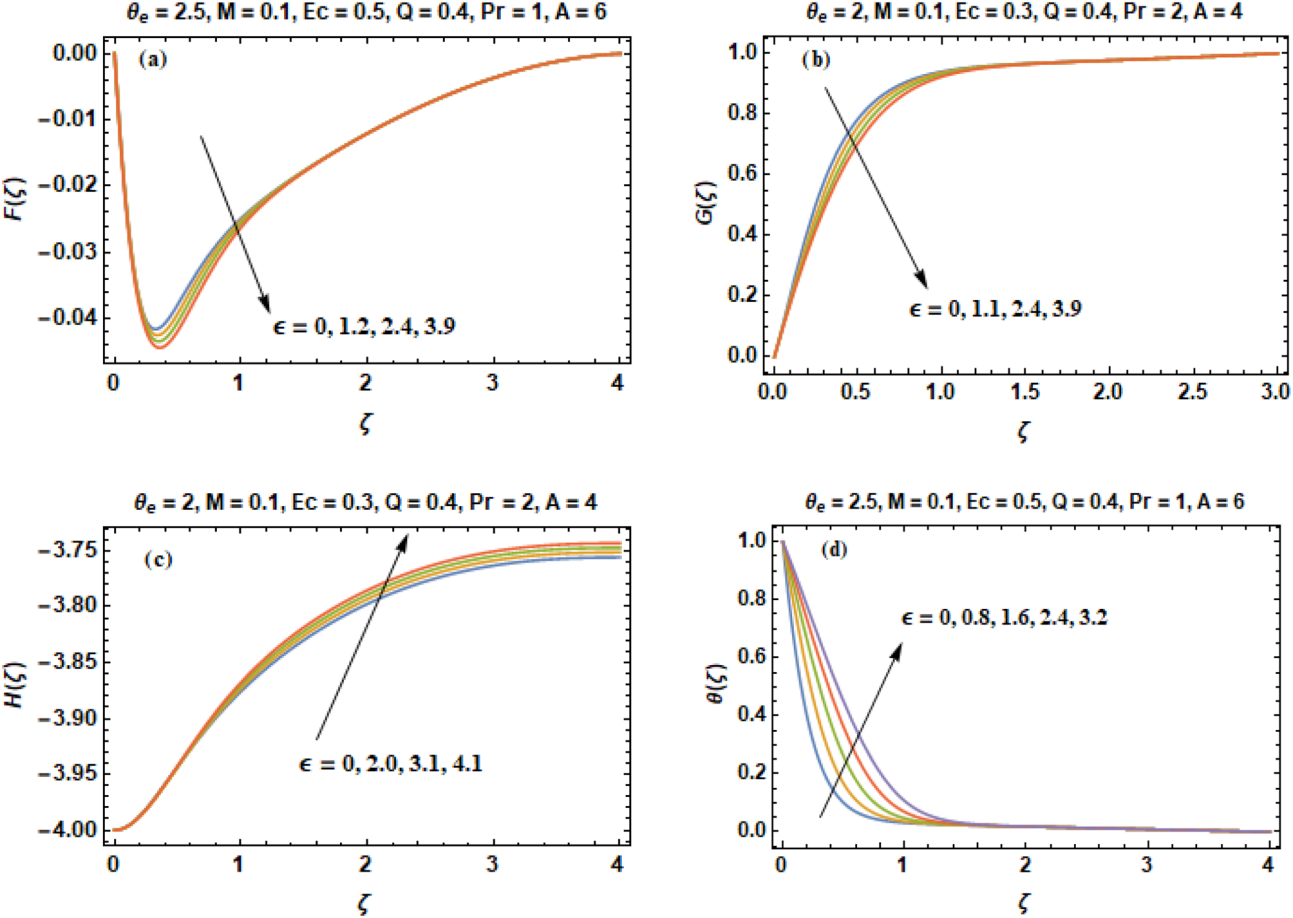
(**a–d**) Components of velocity **(F, G, H)** & temperature profile **(θ)** versus parameter of variable thermal conductivity **(**$\varepsilon$**)**.

Figure 8a–d illustrates the change in velocity constituents and temperature curves for distinct values of parameter of variable thermal conductivity ($\varepsilon$).Thermal conductivity is one of the thermo physical property. Thermal conductivity of a material is defined as the measure of its ability to conduct/transfer heat. Heat is transferred at a higher rate in materials of high thermal conductivity as compared to the materials which have low thermal conductivity. For example, metals are very effective at conducting heat because they have typically high thermal conductivity, while the opposite is correct for insulating materials such as Styrofoam. That’s why we use metal utensils in cooking to allow heat to move through it quickly and around what we are cooking. Figure 8c shows that volumetric flow rate and axial velocity are decreasing functions of thermal conductivity parameter ($\varepsilon$). Such decrease in the axial flow intensity is indemnified by an increment in the inward radial velocity near the disk as illustrated in Fig. 8a. A cross over in the curves of velocity constituent G shown in Fig. 8b revealing that azimuthal velocity (G) increases away from the disk and decreases near the disk when parameter ($\varepsilon$) increases. Figure 8d shows that temperature profiles (θ) becomes thicker when we change the values of parameter ($\varepsilon$). It is because of this reason that thermal conductivity is in direct proportion with the values of $\varepsilon$. Hence an increment in the values of parameter ($\varepsilon$) enhances the thermal condition of fluid that in result thickens the layer of thermal boundary.

**Figure 9:**
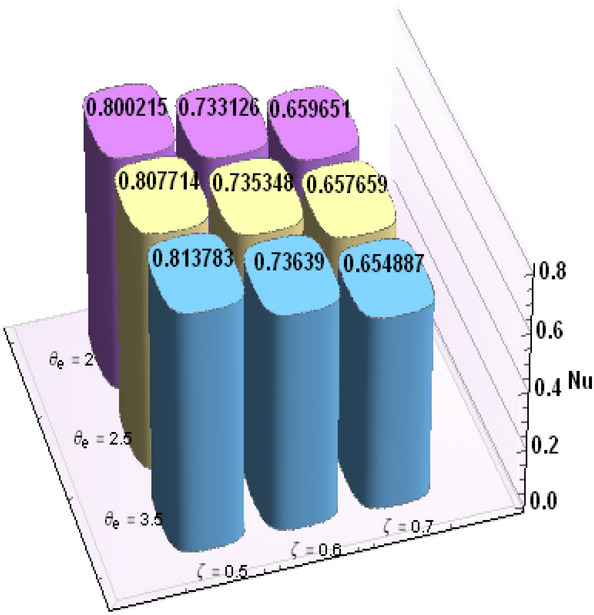
Bar chart representation of Skin friction coefficient and Nusselt number for different parameters using Mathematica 10 software available at (https://softasm.com/wolfram-mathematica-10-4-full-crack/). Nu versus $\theta_{e}$ .

**Figure 10:**
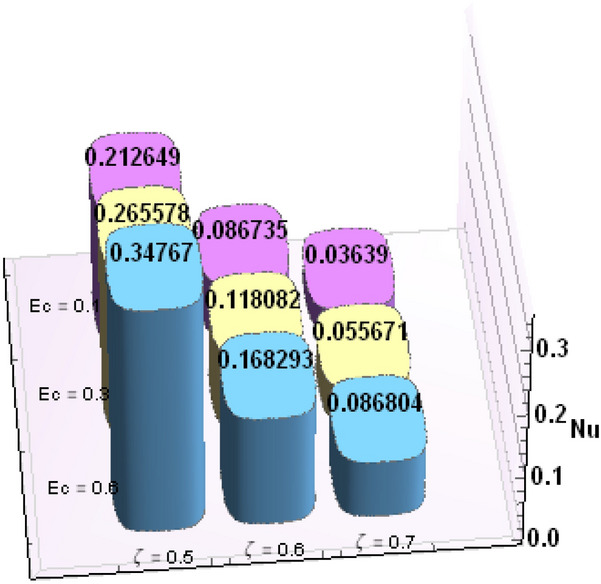
Bar chart representation of Skin friction coefficient and Nusselt number for different parameters using Mathematica 10 software available at (https://softasm.com/wolfram-mathematica-10-4-full-crack/). Nu versus Ec.

**Figure 11:**
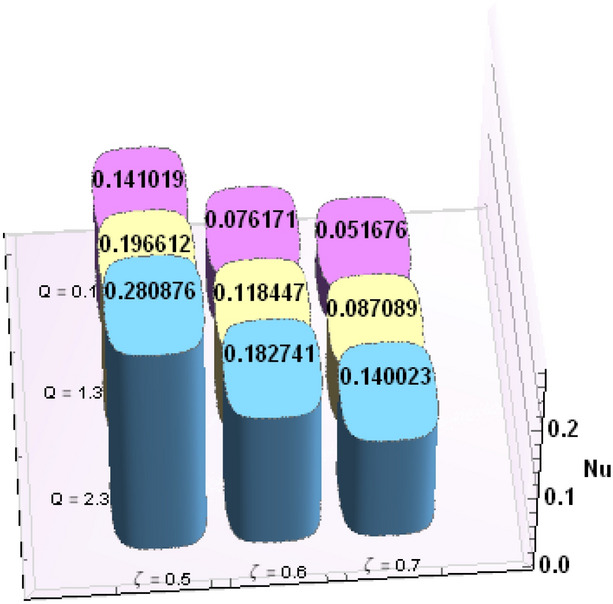
Bar chart representation of Skin friction coefficient and Nusselt number for different parameters using Mathematica 10 software available at (https://softasm.com/wolfram-mathematica-10-4-full-crack/). Nu versus Q.

**Figure 12:**
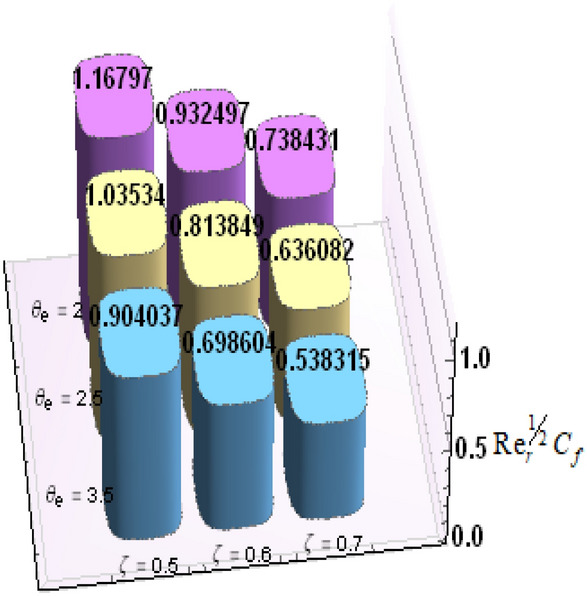
Bar chart representation of Skin friction coefficient and Nusselt number for different parameters using Mathematica 10 software available at (https://softasm.com/wolfram-mathematica-10-4-full-crack/). $${\text{Re}}_{r}^{{{\raise0.7ex\hbox{$1$} \!\mathord{\left/ {\vphantom {1 2}}\right.\kern-\nulldelimiterspace} \!\lower0.7ex\hbox{$2$}}}} C_{f}$$ versus $$\theta_{e}$$.

**Figure 13:**
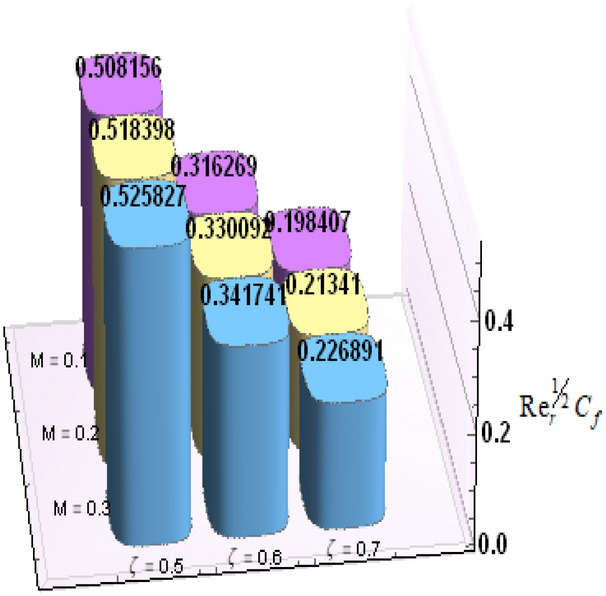
Bar chart representation of Skin friction coefficient and Nusselt number for different parameters using Mathematica 10 software available at (https://softasm.com/wolfram-mathematica-10-4-full-crack/). $${\text{Re}}_{r}^{{{\raise0.7ex\hbox{$1$} \!\mathord{\left/ {\vphantom {1 2}}\right.\kern-\nulldelimiterspace} \!\lower0.7ex\hbox{$2$}}}} C_{f}$$ versus M.

**Figure 14:**
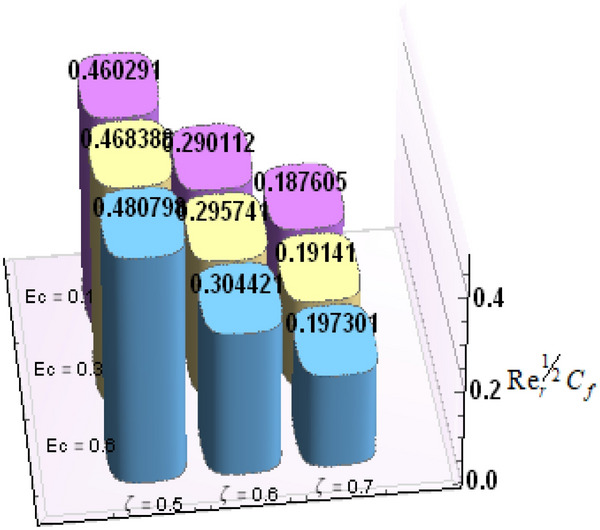
Bar chart representation of Skin friction coefficient and Nusselt number for different parameters using Mathematica 10 software available at (https://softasm.com/wolfram-mathematica-10-4-full-crack/). $${\text{Re}}_{r}^{{{\raise0.7ex\hbox{$1$} \!\mathord{\left/ {\vphantom {1 2}}\right.\kern-\nulldelimiterspace} \!\lower0.7ex\hbox{$2$}}}} C_{f}$$ versus Ec.

**Figure 15:**
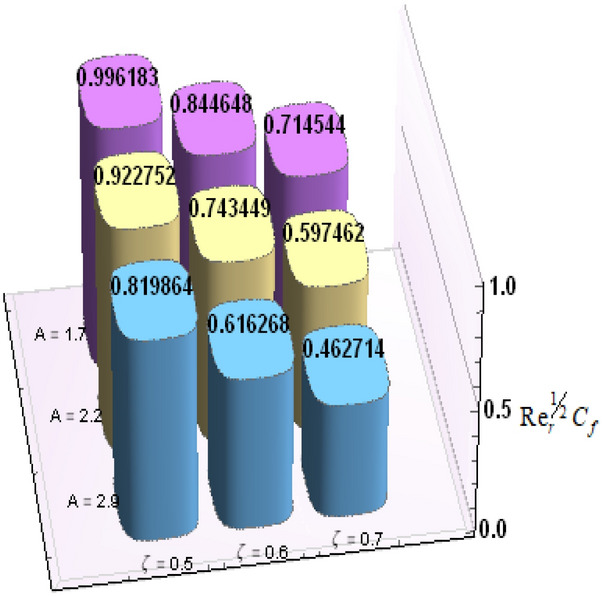
Bar chart representation of Skin friction coefficient and Nusselt number for different parameters using Mathematica 10 software available at (https://softasm.com/wolfram-mathematica-10-4-full-crack/). $${\text{Re}}_{r}^{{{\raise0.7ex\hbox{$1$} \!\mathord{\left/ {\vphantom {1 2}}\right.\kern-\nulldelimiterspace} \!\lower0.7ex\hbox{$2$}}}} C_{f}$$ versus A.

**Figure 16:**
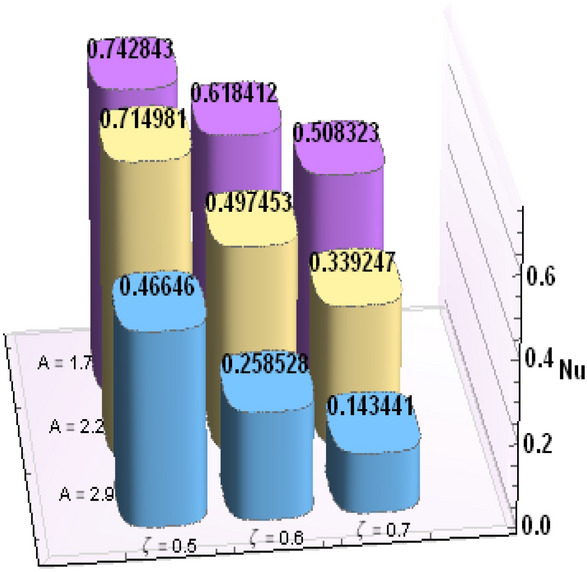
Bar chart representation of Skin friction coefficient and Nusselt number for different parameters using Mathematica 10 software available at (https://softasm.com/wolfram-mathematica-10-4-full-crack/). Nu versus A.

**Figure 17:**
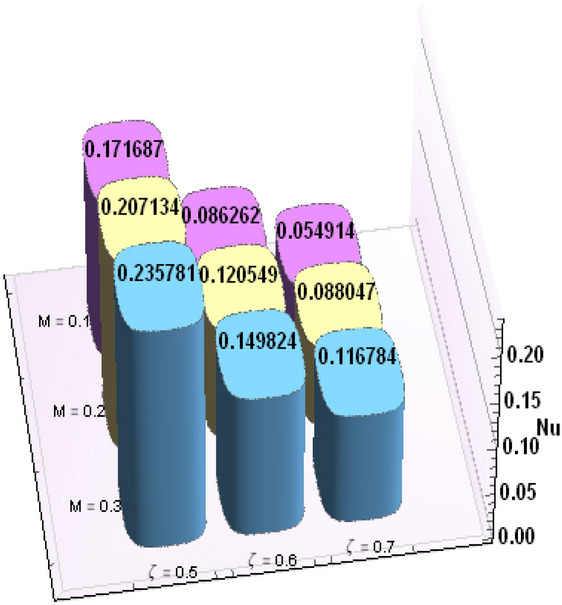
Bar chart representation of Skin friction coefficient and Nusselt number for different parameters using Mathematica 10 software available at (https://softasm.com/wolfram-mathematica-10-4-full-crack/). Nu versus M.

A comparative analysis has been carried out and results are displayed in Table. 1. A very good agreement is observed between existing results and those of Rafiq et al. ^53^. Results of skin friction parameter and Nusselt number for distinct values of emerging parameters are presented in Tables 2 and 3. Further, bar charts displayed the tabular data of skin friction parameter and nusselt number for various parameters in Figs. 9, 10, 11, 12, 13, 14, 15, 16 and 17. Table 2 demonstrate the impact of $A, \, \varepsilon , \, Q, \, Ec, \, M{\text{ and }}\theta_{e}$ against skin friction parameter. In boundary layer flows, the coefficient of skin friction is a crucial non-dimensional parameter. It specifies the fraction of the local dynamic pressure, $\frac{1}{2}\rho U^{2}$, that is felt as shear stress on the surface. Table 2 shows that value of skin friction parameter decreases when we increase the value of A and $\theta_{e}$ whereas skin friction parameter increases when we increase the value of $\varepsilon , \, Q,Ec{\text{ and }}M$ . Table 3 highlighted the impact of $A, \, \varepsilon , \, Q, \, Ec, \, M{\text{ and }}\theta_{e}$ on (Nu). In fluid dynamics, the nusselt number is defined as the ratio of convective heat transfer to conductive heat transfer across a boundary in a fluid. Convection comprises of both diffusion (conduction) and advection (motion of fluid). When Nu = 1, it means that the heat transfer by pure conduction or we can say that fluid is stationary. The value of nusselt number between 1 & 10 is a feature of laminar flow or slug flow. The greater value of nusselt number corresponds to more active convection, with turbulent flow usually in the 100- 1000 range. It is noticed that an increment in magnitude of A decays nusselt number while a direct trend is noticed for $\varepsilon , \, Q, \, Ec, \, M{\text{ and }}\theta_{e}$.

**Table 1:** Effects of parameter of wall suction A on wall shear stresses $F^{\prime}(0)$& $G^{\prime}(0)$ when Pr = 5, $\theta_{e} = 10000, \, \varepsilon = 1.$ Comparison of current results with those of Rafiq et al. ^51^.

	$- F^{\prime}(0)$			$G^{\prime}(0)$		
A	Rafiq et al. ^51^	Present results	Difference	Rafiq et al. ^51^	Present results	Difference
0	0.941924	0.949791	− 0.007867	0.772847	0.783231	− 0.010384
0.5	0.903232	0.916672	− 0.01344	1.065148	1.0776	− 0.012452
1	0.835092	0.835262	− 0.00017	1.388375	1.39276	− 0.004385
2	0.646787	0.646763	0.000024	2.155786	2.15579	− 0.000004
3	0.479982	0.479983	− 0.000001	3.060799	3.0608	− 0.000001

**Table 2:** Numerical values of Skin friction coefficient $${\text{Re}}_{r}^{{{\raise0.7ex\hbox{$1$} \!\mathord{\left/ {\vphantom {1 2}}\right.\kern-\nulldelimiterspace} \!\lower0.7ex\hbox{$2$}}}} C_{f}$$ against several physical quantities.

A	Pr	ε	Q	Ec	M	$$\theta_{e}$$	$${\text{Re}}_{r}^{{{\raise0.7ex\hbox{$1$} \!\mathord{\left/ {\vphantom {1 2}}\right.\kern-\nulldelimiterspace} \!\lower0.7ex\hbox{$2$}}}} C_{f}$$[0.5]	$${\text{Re}}_{r}^{{{\raise0.7ex\hbox{$1$} \!\mathord{\left/ {\vphantom {1 2}}\right.\kern-\nulldelimiterspace} \!\lower0.7ex\hbox{$2$}}}} C_{f}$$[0.6]	$${\text{Re}}_{r}^{{{\raise0.7ex\hbox{$1$} \!\mathord{\left/ {\vphantom {1 2}}\right.\kern-\nulldelimiterspace} \!\lower0.7ex\hbox{$2$}}}} C_{f}$$[0.7]
1.7	3.0	1.0	0.2	0.3	0.1	3.0	0.99618	0.84464	0.71454
2.2	3.0	1.0	0.2	0.3	0.1	3.0	0.92275	0.74344	0.59746
2.9	3.0	1.0	0.2	0.3	0.1	3.0	0.81986	0.61626	0.46271
6.0	1.0	0.2	0.4	0.5	0.1	2.5	0.48244	0.27581	0.15946
6.0	1.0	0.8	0.4	0.5	0.1	2.5	0.53268	0.30697	0.17785
6.0	1.0	1.6	0.4	0.5	0.1	2.5	0.59541	0.34909	0.20411
5.0	5.0	1.0	0.1	1.0	0.2	2.0	0.58866	0.37622	0.24477
5.0	5.0	1.0	1.3	1.0	0.2	2.0	0.62915	0.40536	0.26512
5.0	5.0	1.0	2.3	1.0	0.2	2.0	0.68613	0.44681	0.29429
5.0	2.0	1.0	0.4	0.1	0.2	5.0	0.46029	0.29011	0.18760
5.0	2.0	1.0	0.4	0.3	0.2	5.0	0.46838	0.29574	0.19141
5.0	2.0	1.0	0.4	0.6	0.2	5.0	0.48079	0.30442	0.19730
5.0	5.0	1.0	0.4	1.5	0.1	4.0	0.50815	0.31626	0.19840
5.0	5.0	1.0	0.4	1.5	0.2	4.0	0.51839	0.33009	0.21341
5.0	5.0	1.0	0.4	1.5	0.3	4.0	0.52582	0.34174	0.22689
3.0	1.0	1.0	0.4	0.5	0.1	2.0	1.16797	0.93249	0.73843
3.0	1.0	1.0	0.4	0.5	0.1	2.5	1.03534	0.81384	0.63608
3.0	1.0	1.0	0.4	0.5	0.1	3.5	0.90403	0.69860	0.53831

**Table 3:** Numerical values of Nusselt number Nu against several physical quantities.

A	Pr	ε	Q	Ec	M	$\theta_{e}$	Nu [0.5]	Nu [0.6]	Nu [0.7]
1.7	3.0	1.0	0.2	0.3	0.1	3.0	0.74284	0.61841	0.50832
2.2	3.0	1.0	0.2	0.3	0.1	3.0	0.71498	0.49745	0.33924
2.9	3.0	1.0	0.2	0.3	0.1	3.0	0.46646	0.25852	0.14344
6.0	1.0	0.2	0.4	0.5	0.1	2.5	0.47184	0.27443	0.15924
6.0	1.0	0.8	0.4	0.5	0.1	2.5	0.70025	0.43585	0.26279
6.0	1.0	1.6	0.4	0.5	0.1	2.5	0.95422	0.68563	0.46178
5.0	5.0	1.0	0.1	1.0	0.2	2.0	0.14101	0.07617	0.05167
5.0	5.0	1.0	1.3	1.0	0.2	2.0	0.19661	0.11844	0.08708
5.0	5.0	1.0	2.3	1.0	0.2	2.0	0.28087	0.18274	0.14002
5.0	2.0	1.0	0.4	0.1	0.2	5.0	0.21264	0.08673	0.03639
5.0	2.0	1.0	0.4	0.3	0.2	5.0	0.26557	0.11808	0.05567
5.0	2.0	1.0	0.4	0.6	0.2	5.0	0.34767	0.16829	0.08680
5.0	5.0	1.0	0.4	1.5	0.1	4.0	0.17168	0.08626	0.05491
5.0	5.0	1.0	0.4	1.5	0.2	4.0	0.20713	0.12054	0.08804
5.0	5.0	1.0	0.4	1.5	0.3	4.0	0.23578	0.14982	0.11678
3.0	1.0	1.0	0.4	0.5	0.1	2.0	0.80021	0.73312	0.65965
3.0	1.0	1.0	0.4	0.5	0.1	2.5	0.80771	0.73534	0.65765
3.0	1.0	1.0	0.4	0.5	0.1	3.5	0.81378	0.73639	0.65488

## Conclusions

In the current study a mathematical model has been presented for the influences of heat transport in a Bodewadt flow in the existence of viscous dissipation, wall suction, magnetic field, heat generation/absorption, joule heating and variable characteristics of fluid. The consequence of the current problem demonstrated that appropriate amount of wall suction is essential to restore well matched solutions of thermal energy equation physically. The key points of the current effort are appended as follows:

- The magnetic field (M) shows the opposite impact on velocity profile which means the velocity of the fluid diminishes with an increment in the value of M. Reason is that the Lorentz forces are against the fluid flow. The temperature profiles improves with an increment in magnetic field.
- Opposite behavior of heat generation/absorption effects is observed over profile of temperature.
- Boundary layer is substantially thinned in Bodewadt’s flow owing to the insertion of wall suction. All constituents of velocity & temperature are diminishing functions of variable viscosity parameter $\theta_{e}$. Moreover, when higher $\theta_{e}$ is employed than the transformation in solution profiles with an increment in suction decreases.
- When parameter of variable thermal conductivity ε becomes greater, than deterioration in rate of heat transfer is observed.
- Temperature increases against higher values of Eckert number Ec.

## Data Availability

The datasets used and/or analyzed during the current study available from the corresponding author (A. A. or K. S. N.) on reasonable request.

## List of symbols

$\left( {V_{r} , \, V_{\varphi } , \, V_{z} } \right)$: Radial, circumferential and axial velocity component(*ms-1*)
$\left( {{\text{r, }}\varphi {\text{, z}}} \right)$: The coordinate system
T: Temperature of the fluid
$$T_{\infty }$$: Ambient temperature
$T_{w}$: Temperature of the disk
$C_{p}$: Specific heat capacity(*Jkg*^*-1*^* K*^*-1*^)
$\mu_{\infty }$: Ambient viscosity of the fluid
$B_{0}$: Magnetic field intensity
$Q^{*}$: Heat generation/absorption parameter
$K_{\infty }$: Ambient thermal conductivity
$\theta_{e}$: Parameter of variable viscosity
Pr: Prandtl number
A: Wall suction parameter
M: Magnetic field parameter
Ec: Eckert number
$\tau_{r}$: Radial wall stress
$\tau_{\varphi }$: Circumferential wall stress

## Greek symbol

$\kappa$: Thermal conductivity(*Wm*^*-1*^* K*^*-1*^)
$$\rho$$: Density(*kgm*^*-3*^)
$\mu$: Dynamic viscosity(*kgm*^*-1*^* s*^*-1*^)
$\sigma$: Electrical conductivity of the fluid
$\varepsilon$: Temperature ratio parameter

## Subscript

*w*: Wall condition
*e*: Free stream
∞: Ambient condition
